# “COVID-19 affected me greatly (sigh), imagine I'm being called a mother and yet I'm also a child”: the effect of COVID-19 on fertility management practices among women in Nairobi and Kisumu cities, Kenya

**DOI:** 10.3389/fgwh.2025.1428133

**Published:** 2025-02-24

**Authors:** Zachary Arochi Kwena, Pauline Wekesa, Serah Gitome, Sarah Okumu, Louisa Ndunyu, Elizabeth Bukusi, Emily Himes, Kelsey Holt, Jenny Liu, Janelli Vallin, Lauren Suchman

**Affiliations:** ^1^ Centre for Microbiology Research, Kenya Medical Research Institute, Nairobi, Kenya; ^2^ School of Public Health, Maseno University, Kisumu, Kenya; ^3^ Social and Behavioral Sciences, University of California, San Francisco, CA, United States

**Keywords:** fertility management practices, COVID-19 containment measures, contraception, healthcare services, Kenya

## Abstract

**Background:**

The COVID-19 pandemic strained the provision of sexual and reproductive health services, including family planning, which were categorized as non-essential services at the peak of COVID-19 infection control in Kenya. We set out to assess the effect of COVID-19 on fertility management practices among Kenyan women in two cities to inform mitigation measures in future similar disruptions.

**Methods:**

This was a qualitative study to describe the effect of the COVID-19 pandemic on women's fertility management practices from 61 in-depth interviews (IDIs) with women aged 15–45 years residing in Nairobi and Kisumu, Kenya, between February and May 2021. Identified participants were consented and interviewed at convenient locations. We used a constant comparative analysis that compared emergent themes across topics and transcripts to identify and group those that are similar or dissimilar to arrive at insights that informed our conclusions.

**Results:**

Our findings show that COVID-19 affected women's fertility management practices at individual, interpersonal, and organizational levels. At the individual level, lack of money due to COVID-19-induced economic difficulties made family planning services unaffordable to women. Other women delayed their conception plans until their financial situation improved. At the interpersonal level, travel restrictions separated couples, making it challenging for those who had plans to conceive to actualize their fertility plans. Additionally, women who reported to be sexually inactive relaxed adherence to their contraceptive use schedule because of the reduced risk of unintended pregnancy. Finally, at the organizational level, provider shortages, long queues, and contraceptive stockouts during COVID-19 compromised women's access to family planning services. We also found that a minority of women who were either not using contraceptives or who were on long-acting methods perceived little or no effect of COVID-19 on their fertility management practices.

**Conclusion:**

COVID-19's effect on women's fertility management practices manifested at individual, interpersonal, and organizational levels. There is a need to devise strategies that empower women to deal with their family planning needs and those that make healthcare systems resilient enough to handle the effects of emergent crises without compromising the provision of existing services.

## Introduction

COVID-19 disease, caused by the SARS-CoV-2 virus, rapidly spread to almost all countries, infecting millions of individuals and resulting in numerous deaths. This global pandemic slowed and even shut down many sectors of the economy, including health. Among the health services affected were sexual and reproductive health (SRH) (1). Like many other sub-Saharan Africa (SSA) countries with inherently weak and low resilient systems, Kenya bore the brunt of the disease that strained the already under-resourced and ill-equipped systems (2, 3). Services such as family planning were disrupted, thereby affecting the continuation of women's fertility management, which is paramount to avoiding long-term, unintended consequences to the well-being of individuals and families (4).

As a response to COVID-19, the Kenyan government instituted a raft of public health interventions to interrupt its transmission in the population (5). The measures mostly targeted reducing crowding and movement of people that was associated with the “moving virus” in the process. Specifically, the measures included a ban on international travel, closure of school and other learning institutions, ban on social gatherings and meetings, a dawn to dusk curfew, closure of religious places, bars, and restaurants, and observance of physical distancing in spaces where people gather (6). Others were the directive to focus on offering essential services and encouraging people to work from home. All these measures led to service disruptions in many sectors.

Family planning was among the services documented to have been most affected by the COVID-19 pandemic in many countries in sub-Saharan Africa (7, 8). This included associated support resources such as financial and human that were temporarily redeployed to handle COVID-19 emergencies (8). Indeed Kenya was among the 68% of sub-Saharan African countries that reported COVID-19 and containment measures to have significantly affected family planning services (9). The decision to categorize family planning as non-essential and bundle it together with maternal health services worsened the situation (9). Consequently, women's fertility management practices, the way women implement or act on their fertility plans and desires, were affected either because of necessity—when women couldn't access contraception, or out of preference—when women didn't want to access contraception even though they still didn't want to become pregnant.

Undoubtedly, COVID-19-related disruptions placed some women at increased risk of unintended pregnancies, usually associated with increased maternal, newborn, and child morbidity and mortality and other nonmedical problems (10, 11). For instance, a study among secondary school girls in western Kenya shows that girls experiencing COVID-19 containment measures had twice the risk of falling pregnant (55/509, 11%) before completing secondary relative to girls (21/403, 5%) in the pre-COVID-19 period (12) Another study in the same region showed that adolescent girls and young women (AGYW) had 60% increased risk of being pregnant during the lockdown when compared to pre-lockdown period (13). The continued disruptions to contraceptive access, either due to COVID-19 or other emergencies, deny women their reproductive decision-making rights and autonomy thereby eroding the gains achieved in SRH over the years (14). Thus, while it is apparent that the COVID-19 pandemic affected women's fertility management practices, the extent and nature of these effects (whether they were supply or demand side) are not clearly known, especially in the context of countries like Kenya with documented weaknesses in the healthcare systems. Thus, taking a socio-ecological approach (15), we set out to assess the extent and nature of the effect of COVID-19 on women's fertility management practices to inform mitigation measures to avert disruptions from similar emergencies in future.

## Methods

### Study design

This was a cross-sectional qualitative study to assess the effect of the COVID-19 pandemic on women's fertility management practices using a socio-ecological framework, which recognizes that human behavior and health outcomes are influenced by multiple levels of factors ranging from individual to broader societal factors (16, 17). This paper draws data from 61 in-depth interviews conducted between February and May 2021 in Nairobi and Kisumu metropolitan areas in Kenya.

### Study settings and population

This study was conducted as part of the Innovations in Choice and Autonomy (ICAN) project launched in late 2019 and was implemented during the height of the COVID-19 pandemic. Though the ICAN project had a particular focus on understanding preferred models of implementation for supporting self-injection of subcutaneous depot medroxyprogesterone acetate (DMPA-SC), we were also able to gather data on how women's contraceptive use and plans were affected by COVID. Our study population consisted of women of reproductive potential aged 15–45 residing in metropolitan areas of Nairobi and Kisumu. Nairobi and Kisumu both have city status and are located in central and western Kenya, respectively. They have a cumulative population of over approximately six million inhabitants (18). Unlike in Nairobi (6%), most health facilities in Kisumu (60%) are public (19, 20). In Nairobi, the contraceptive prevalence among women of reproductive age is 58% as compared to 53% nationally. The unmet needs for family planning amongst the urban poor remain a big challenge due to the question of commodity accessibility and affordability (21, 22).

### Sampling and sample size

We determined the sample size of 61 women to be sufficient to reach theoretical saturation of the main themes, including the effect of COVID-19 on women's fertility management practices, which we were investigating based on Guest and colleagues' guidelines of determining sample size for in-depth interviews (23). Women were purposively sampled based on their age, marital status, prior contraceptive use or non-use, and previous experience with DMPA-SC. Our inclusion criteria were (1) being aged between 15 and 45, (2) sexually active, (3) residing with Nairobi or Kisumu metropolitan areas, and (4) being willing and able to give consent or assent. Most participants were recruited in the community after being identified through the community health volunteers. In the age category, our sample was divided between age groups 15–19 years and ages 20–45 years because adolescents often have different attitudes toward and experiences with contraception than older, often married, women.

### Data collection

Trained and experienced female qualitative research assistants (RAs) conducted the interviews in a private, mutually agreed upon location, either at the research offices or in the community. The in-depth interview guides used were developed in English, translated into Dholuo and Kiswahili, and then back-translated into English to ensure translation accuracy. At the time of the interview, consenting and assenting participants were interviewed for approximately 60 min in a private room with a trained and experienced qualitative interviewer in a language of their choice. Participants were allowed to have a break during the interview if they needed it. Data collection followed simplified grounded theory lite approaches where additional topics and different categories of participants were pursued based on the emerging data from simplified grounded theory analysis (24). The use of these approaches was supported by the need to uncover novel insights and build contextually relevant frameworks in relatively new COVID-19 literature. Among other topics, participants were asked if COVID-19 influenced their thoughts about pregnancy and actual access to contraceptives. Follow-up and probe questions were guided as much as possible by the participants themselves, and we allowed participants to determine the pace and content of the interview. This method allowed for new questions to emerge during the interviews (25, 26) thus giving us a thorough view and rich narratives into the participant's experiences and perspectives about their fertility management practice during COVID-19. With permission, the interviews were audio-recorded and promptly downloaded into password-protected folders on a study computer.

### Data analysis

Audio files were transcribed into English by experienced transcriptionists, and the resultant transcripts were checked for quality before they were approved and uploaded to Dedoose (Sociocultural Research Consultants, LLC) for coding. Data coding and analysis was guided by socio-ecological framework and conducted concurrently with data collection. We familiarized ourselves with the emerging data by reading the transcripts and noting the initial ideas we used in developing the codebook. We used a combination of deductive and inductive approaches to develop a codebook, which included identifying potential codes from the interview guides as well as from the available transcripts. We used the scrutiny techniques proposed by Ryan and Bernard to identify codes and subcodes by paying attention to similarities and differences within and across transcripts (27). Eight coders, paired for the purposes of double coding, were involved in coding to determine the code application consistency and refine the codebook as necessary (28). The discrepancies among the coders were discussed in scheduled meetings to reach a consensus which sometimes included further refining the definition of codes and subcodes in the codebook. Relevant data excerpts, together with memos that contained analytic notations that contextualized and described emerging key findings, were extracted, and typical statements were used for citations. We used the constant comparative analysis method to discover dominant themes by comparing and contrasting within and between transcripts, study sites, age categories, marital and contraception use (29). The constant comparative analysis enabled us to form categories, establish the boundaries of the categories, assign the segments to categories, summarize the content of each category and find positive or negative evidence of the effect of COVID-19 on fertility management practices.

## Results

### Socio-demographic characteristics

Table 1 shows the characteristics of the women we interviewed. In summary, about 60% were in the category of adolescent girls and young women, with about two-thirds (66%) having post-primary education. Similarly, another two-thirds of the women reported being single (62%) and unemployed (64%). More than half reported having children (57%) and being on contraceptives (59%) (Table 1).

**Table 1:** Socio-demographic characteristics of women enrolled in the study.

Attribute	Count	Percent
Age		
15–24	36	59
25–49	25	41
Education		
Primary	20	34
Post primary	38	66
Marital status		
Single	39	62
Married	22	34
Children		
No children	26	43
Has children	35	57
Employment		
Employed	22	36
Not employed	39	64
Contraceptive use		
No	25	41
Yes	36	59

### Organizational level

At an organizational level, participants identified provider shortages, long queues, reduced access to providers due to prioritization of COVID-19-related cases, and commodity stockouts as factors that affected women initiating or continuing with contraception. The government directive for elderly workers to work from home because of their unique vulnerabilities to COVID-19 infection and other staffing rationalizations during this time resulted in provider shortages even at the health facilities. As such, it was not uncommon to observe long queues at the health facilities due to fewer providers attending to women seeking SRH services which deterred women from seeking these services.

COVID-19 has affected because in government hospitals, they don't allow many people providers. and the women who have that need contraception are many (Aged 32, married with 4 children, Nairobi).

Additionally, COVID-19 care was prioritized over other services, such as SRH, that were categorized as non-essential services. This effectively led to the redeployment of providers who ordinarily attend to patients seeking contraception services to other areas such as maternal and child health that were considered essential, thus compromising the quality of care for women seeking contraception services.

Your family planning appointment date has come, and the doctors are also saying that…they don't want many people in there as they are attending to young babies (Aged 32 married with 4 children, Nairobi)

Commodity stockout was another supply-side factor that affected women's fertility management practices. Overall, the COVID-19 period was characterized by stockouts blamed on COVID-19 containment measures that slowed down transportation of commodities to health facilities. Thus, some participants reported that they went for their contraception refill only to be informed that their method was out of stock.

… the doctors are also saying that the medicines have not arrived… yet at that time, you don't even have money to go to a private hospital, so if you don't know how you will protect yourself, you will find yourself conceiving without expecting (Aged 32, married with 4 children, Nairobi)

Some participants reported countering organizational barriers to access to contraception by shifting to receive their family planning services at private health facilities, even though this came at a higher cost.

### Effect at interpersonal level

Lockdowns and travel restrictions instituted as COVID-19 containment measures separated sexual partners who, at the time of COVID-19, were living in different locations due to work or other reasons. These measures made it difficult for partners to travel and physically meet for coitus which was reported to affect women's fertility management, especially those who were married and desired to conceive.

I can say like now there is lockdown I can't go home the father to my children is also home and I am here he cannot come here neither can I go there just that…Who will I get pregnant with? (laughter) (Aged 32, married with 2 children, Nairobi)

On the other hand, some women were fearful of meeting their partners in case their partners were infected and could transmit the virus to them.

I can't travel much like going and seeing my boyfriend because I'm also afraid of the Covid because I don't know the people he is around with, I don't know where he goes, I don't know what he does maybe he can bring Covid when we meet, I can be affected I don't want that to happen (Aged 17, married with no child, Nairobi).

Some women reported relaxing their contraceptive use during COVID-19, arguing that they were not sexually active at the time. Some reported stopping contraceptive use altogether, saying that they did not see the need since they were sexually inactive.

… like I said, people are not seeing each other so… I am not frequently having sex and I do not need the contraceptives as much right now (Aged 25, single with 1 child, Nairobi)

Due to uncertainties, other participants reported thoughts of abstaining from sex altogether until the COVID-19 situation improved.

With this COVID you can…I can decide together to abstain from sex until maybe things go well, until the pandemic goes down, yes (Aged 25, single with no child, Nairobi).

### Effect at personal level

Diminished cash flow due to business closure and employee layoff and reduced pay for the few retained in employment made family planning methods unaffordable for some women. In addition to diminished cash flow, contraceptive providers such as private hospitals and retail pharmacies increased commodity prices supposedly due to high running costs coupled with high demand for the commodities. This constellation of situations compromised access and made some women to abandon contraception use altogether. For instance, one woman whose contraceptive review date had come and didn't have the money required said:

…if you look at it keenly, women have been trapped during this COVID-19 pandemic… your appointment date has come and at that time I didn't have money because there was no going to work and my husband also didn't have money because he could go for his casual jobs and bring back something small (Aged 32, married with 4 children, Nairobi)

In other circumstances, some women reported delaying their pregnancy plans until such a time that they would feel more financially secure. Otherwise, women were concerned about how they would deal with inflation to provide for their babies if they were to conceive.

You have to get something to put on the table was also a problem. so, you might look at that figure and you say there is no need of maybe having a child at this time because how you will be feeding them might be a problem (Aged 34 married with 3 children, Kisumu).

This financial uncertainty made a number of women to shift from the methods that they were on to more effective methods to further reduce the risk of unintended pregnancy. Actually, some women who were less confident with the methods that they were on ended up switching to what they considered to be more effective methods to ensure that they did not become pregnant during COVID-19.

“So, I'm planning to look for another alternative method of family planning to use that will be suitable. Yeah, because I don't want to get pregnant right now” (Aged 21, married with 2 children, Nairobi).

Some women also delayed their pregnancy for fear of transmitting COVID to their babies during birth and breastfeeding period. Further, participants also argued that pregnancy and childbirth weaken the mothers' immunity making them vulnerable to catching COVID-19 and likely transmitting to their babies because of their weak immune system.

The only thing I fear is going to labor when COVID-19 is still present, so I fear it might infect my baby. That is the only thing that I fear (Aged 17 married with no children, Nairobi).

…if I were to get pregnant now that COVID-19 is there, it would really affect me, and it would give me a hard time…You know when I have given birth, there is no way I will maintain social distance from the baby, obviously I will have to keep the baby close plus a baby can't wear a mask, the little one cannot be protected… you know the baby's immunity is weak, so if the baby gets Covid, its survival chances are very minimal (Aged 19 single with no children, Kisumu).

Another effect of COVID-19 on fertility management practices was exhibited in adolescent girls in school using contraceptives covertly who found it hard to have a convincing reason to leave home to secretly access contraceptives. This inability to access contraceptive services covertly might have altered the girls' fertility management practices, resulting in an upsurge of teenage pregnancies among schoolgirls during prolonged government school closure. The prolonged closure of schools coupled with people working from home seemed to have exposed schoolgirls to the risk of pregnancy, especially from immediate neighbours in informal settlements. One young participant lamented that she became pregnant at a young age due to lack of contraceptive access during the pandemic.

“COVID-19 affected me greatly (sigh) imagine I'm being called a mother and yet I'm also a child. I have been called a mother at a very young age” (Aged 17, single with 1 child, Nairobi).

However, a few women receiving their family planning services within or close to their residential areas were not affected by COVID-19. This is because they could still reach their facilities without breaking the restriction directives. Specifically, these women reported that they could quickly dash to their providers and return to their houses and, as such, they still had access to their contraceptive products

It has not affected me because I leave the house and. then I go get injected; it's not a meeting (Aged 30, married with 3 children, Kisumu).

Additionally, some women who were on long-acting methods felt no effect of COVID-19 since their refill or review transcended the pandemic period. For instance, women who had initiated their long-acting methods before COVID-19 lived through the era of COVID-19 without the need for contraceptive services at a health facility. When asked how COVID-19 has their contraceptive use practices, one woman answered that:

No, it has not affected me. I put this prevention method even before COVID-19 happened (Aged 38 single with 3 children, Kisumu).

However, other women simply did not see a link between COVID-19 and getting pregnant because to them, these were two different things.

It has not affected because COVID-19 is a disease that affects the breathing system not the reproductive system (Aged 19, single with no child, Nairobi).

“It has not affected me in line with preventing pregnancy because COVID is on its own and pregnancy is also on its own” (Aged 19, single with 1 child, Nairobi).

## Discussion

We set out to assess the extent and nature of the effect of COVID-19 pandemic on women's fertility management practices in Kenya. We found that the effect on *w*omen was at personal, interpersonal, and organizational resulting in switches between methods, delayed pregnancy plans, partners being unable to travel and meet for coitus, and staff shortages and long queues at the health facilities.

Differential effects of the COVID-19 pandemic on women's contraceptive use equally points to the need for differentiated intervention strategies sensitive to the needs of the diverse groups of women. Women in lower socio-economic stratum are likely to be affected more by price increases that made contraceptive commodities relatively unaffordable. This happened during COVID-19 when the government health facilities that offer free or subsidized commodities became either inaccessible or had stockouts, and women had to find their commodities elsewhere, including private-for-profit health facilities. This, undoubtedly, might have upset the fertility plans of vulnerable women from poor socio-economic backgrounds whose unstable financial situations were already exacerbated by effects of COVID-19. Indeed, Zimmerman et al. show that income loss was associated with women from the poorest stratum of society wanting to delay or limit childbearing (30). Unfortunately, this coincided with the time family planning programs that help the women realize their fertility plans were performing dismally. Indicated poor performance of family planning programs during COVID-19 is corroborated by WHO's pulse surveys that showed that among the services most affected were SRH, which caused anxiety to women, especially those who had no immediate intentions of conceiving (3).

Related to switching providers was the need to switch between methods to either use those available in the absence of the most preferred or switch to more effective ones to further prevent any remote risk of conceiving during COVID-19. While the switch between methods is not uncommon, the pandemic presented unique reasons for switching which not only included availability and affordability but also exposure to risk perceptions. Studies in other contexts show that the switches were based on methods that could be used without needing providers' intervention (31, 32). For instance, Walker et al. showed that women switched from contraceptive methods that required face-to-face consultation with a provider such as Implant and intrauterine device to progestogen-only methods to maintain their fertility plans during COVID-19 (31). Similar to our findings, Karp et al. showed that although most women did not change their methods, those who did switched to more effective methods than what they had before the pandemic ostensibly to protect their fertility plans (32).

Covert contraceptive use is common among women in relationships who might not have the autonomy to make decisions about their fertility (33). COVID-19 presented difficult situations for covert users to continue refilling their method supplies without people within their households knowing their contraception use status. This was especially challenging for adolescent girls who were still in school and neither wanted their parents to know about their contraception use status nor be at risk of getting pregnant. Similar to our findings, Kibira et al. report that the fear of accidental disclosure or being discovered to be using contraception during COVID-19 was associated with psycho-social torment that led to method discontinuation (34). This resulted in a bulge of adolescent pregnancy incidences during the long school and college closure as a COVID-19 containment measure. In support of this observation, Zulaika et al. also demonstrated that girls who experienced COVID-19 containment measures had twice the risk of getting pregnant before finishing secondary school and three times the risk of dropping out of school compared to those who didn't experience it (12).

Unknown effects of COVID-19 during pregnancy and on mother-infant pairs thereafter affected some women's fertility management practices. For this reason, some women made deliberate decisions to postpone getting pregnant until they were assured of their antenatal and postnatal outcomes. The women's fears and concerns seem to have been founded, given the anecdotal evidence that pregnant women appear to be at higher risk of morbidity and mortality from COVID-19 compared age-matched nonpregnant women (35–37). A systematic review by Wei and colleagues showed that severe COVID-19 was associated with preeclampsia, preterm birth, gestational diabetes, low birth weight and stillbirth (30), thus supporting women's concerns. Further, a study by Zhu et al. definitively shows that concerns about the effect of COVID-19 on women and fetal health were responsible for women's cancelation of their original fertility plans (38).

Health facility-related factors associated with COVID-19 also affected women's fertility management practices. Directive for older providers to work from home, crowding restrictions, and consideration of SRH as a non-essential service might have kept away women who required initiate or refill their contraception methods, hence exposing them to the risk of unintended pregnancy (39). On the flip side, women who were on methods that required provider's intervention, such as Implant and intrauterine devices, and wanted to discontinue the methods to conceive felt delayed in their plans. Similarly, long waiting times due to provider shortages amidst concerns about the increased risk of infection at the health facilities could have kept away women who required contraception services. Increased waiting time was witnessed in many health facilities across the SSA (40, 41). Some facilities had to devise ways to decongest the clinic, which included initiating tele-health and delivering commodities to the women through existing community structures such as community health volunteers (42–44).

Overall, our findings enabled us to gain knowledge about how different contexts inform decision-making around fertility practices, thus giving us a richer understanding of what motivates women to desire or prevent pregnancies in times of crisis. Additionally, while our study is specific to the COVID-19 pandemic, we could apply these insights to other crisis situations that affect public safety or the accessibility of contraception. Our recommendations to improve family planning service delivery in the context of COVID-19 is centered on being prepared for any outbreak that might strike. This can be achieved through strengthening community contacts, such as community health volunteers, that can aid in distributing or creating new ways to safely deliver contraceptive methods directly to communities during future emergencies. Indeed, Woldie colleagues in a systematic review of studies in sub-Saharan Africa, have demonstrated that services providers by this cadre or health workers were not inferior to those provided mainstream providers and could be further improved by training and support supervision (45). Another option is leveraging wide cell phone coverage to institute tele-consultation and courier of commodities to recipients using a wide network of motorbike taxis. Although this qualitative study is not a direct measure of COVID-19's effect on contraceptive use or unintended pregnancy, it highlights potential pathways in which COVID-19 may directly (e.g., through increased fear of going to the clinic) and indirectly (e.g., through lockdown) result in altered fertility management practices. Displaced fertility plans have a direct association with unintended pregnancies via reduced contraceptive use, increased sex from men being at home, or delayed pregnancies for those with immediate or near-term conception plans.

This study had several limitations. One, we only covered two urban geographic areas, which may have limited the diversity of insights that we obtained from the women, restricting the applicability of our findings to women within urban settings. Two, we failed to seek the perspectives of the healthcare providers to corroborate or rebut women's sentiments about the effect of COVID-19 on their fertility management. Three, the public impact of COVID-19 having been controlled through massive vaccination of people, the applicability of the findings of this study might have been reduced. Despite these limitations, this study still makes important contributions to understanding how other essential services, such as family planning, can be provided even as the health systems tackle emerging pandemics. This is because the findings still apply to a large constituent of urban women as well as offering important insights that can inform planning for other similar pandemic situations in the future.

In conclusion, our study showed that the nature and extent of the effect of COVID-19 on women's fertility management practices was experienced differently at personal, interpersonal, and organizational levels. With the insights of the effect of COVID-19 disaggregated at these levels, targeted mitigation programming through devising strategies that deal with emergent crises without compromising the provision of existing services becomes possible. Future studies may focus on providing information on how devised strategies for dealing with emergent crises can seamlessly be integrated into the existing programs without significantly affecting other services.

## Data Availability

The raw data supporting the conclusions of this article will be made available by the authors, without undue reservation.
